# Supplementary material for: NOVA regulates Dcc alternative splicing during neuronal migration and axon guidance in the spinal cord
Source: eLife. 2016 May 25;5:e14264. doi: 10.7554/eLife.14264 (PMC4930329; doi:10.7554/eLife.14264)
Supplement: Figure 1—source data 1. — DOI: http://dx.doi.org/10.7554/eLife.14264.005 [file elife-14264-fig1-data1.docx]

| **Figure 1-source data 1.** Quantification of neuronal migration and axon projection phenotypes in cultured embryos | | | | |
| --- | --- | --- | --- | --- |
| **siRNA-treated embryos (*Actb-gfp* labeled, Figure 1)** | | | | |
| siRNA | | N (embryos) | N (sections) | % Sections with normal neuronal migration and axon projection |
| Control siRNA | | 6 | 42 | 100 |
| Pan-*Nova* siRNA | | 7 | 43 | 0 |
| **Knockout embryos (*Actb-gfp* labeled, Figure 1)** | | | | |
| Genotype | | N (embryos) | N (sections) | % Sections with normal neuronal migration and axon projection |
| WT | | 7 | 49 | 100 |
| *Nova* dKO | | 3 | 32 | 0 |
| *Dcc* KO | | 4 | 26 | 0 |
| **Rescue of *Nova* dKO embryos (*Actb-gfp* labeled, Figure 8)** | | | | |
| Plasmid | Rescue | N (embryos) | N (sections) | % Sections with restored neuronal migration and axon projection* |
| *gfp* | No | 3 | 32 | 0 |
| *Dcc_long_* | Yes | 5 | 55 | 100 |
| *Dcc_short_* | No | 3 | 36 | 0 |
| *Robo3_A.1_* | No | 3 | 30 | 0 |
| **Knockout embryos (*Atoh1-gfp* labeled, Figure 1-figure supplement 2)** | | | | |
| Genotype | | N (embryos) | N (sections) | % Sections with axons reaching the ventral midline |
| WT | | 7 | 42 | 100 |
| *Nova* dKO | | 3 | 20 | 0 |
| *Dcc* KO | | 3 | 18 | 0 |
| **Rescue of *Nova* dKO embryos (*Atoh1-gfp* labeled, Figure 8-figure supplement 1)** | | | | |
| Plasmid | Rescue | N (embryos) | N (sections) | % Sections with axons reaching the ventral midline |
| *gfp* | No | 3 | 20 | 0 |
| *Dcc_long_* | Yes | 3 | 24 | 96 |
| *Dcc_short_* | No | 3 | 21 | 0 |
| In every case, the phenotype was consistently seen in all embryos examined. All the sections (20μm thick) were collected around the forelimbs. *The rescue of the defects was not 100% complete in individual sections, but was observed in all sections and to comparable levels. WT, wildtype. KO, knockout. dKO, double knockout. | | | | |
